# Books Reviewed

**Published:** 1865-05

**Authors:** 


### BOOKS REVIEWED.

*Lectures on Surgical Pathology, delivered at the Royal College of Surgeons of England.* By James Paget, F. R. S., *Surgeon Extraordinary to Her Majesty the Queen; Surgeon in Ordinary to His Royal Highness the Prince of Wales; Surgeon to St. Bartholomew's and Christ's Hospital. Revised and edited by* William Turner, M. D., *London,* F. R. C. S. E., F. R. S. E., *Senior Demonstrator of Anatomy in the University of Edinburgh. Third American edition. Philadelphia:* Lindsay & Blakiston, 1865.

This work comprises thirty-six Lectures upon Scientific Surgery, which were originally delivered at the Royal College of Surgeons, upon the following subjects: Nutrition—its nature, purpose and conditions; conditions necessary to healthy nutrition; fomative process—growth; hypertrophy; atrophy—degeneration; repair and re-production of injured and lost parts; materials for the repair of injuries; processes for the repair of wounds; repair of fractures; healing of injuries in various tissues; phenomena of inflammation; products of inflammation; developments of lymph; degeneration of lymph; changes produced by inflammation; nature and causes of inflammation; mortification; specific diseases; classification of tumors; simple or barren cysts; compound or proliferous cysts; fatty and fibro-cellular tumors; painful sub-cutaneous tumors; fibrous tumors; cartilaginous tumors; myeloid tumors; osseous tumors; glandular tumors; vascular and recurrent tumors; scirrhus, medullary and epetheheal cancer; melanoid, hæmatoid, ostroid, villous, colloid, and fibrous cancers; pathology of cancers; conditions preceding the cancerous growth; structure and life of the cancerous growths; tubercle. We have copied the headings of chapters that our readers may judge something of the character of the work, and discover that it treats of the fundamental questions in surgical pathology. It is very concise and exact in style, giving the most approved views in pathology, yet not excluding altogether opinions which the author does not adopt. It is a work which should be in the library of every physician—its pages may be perused with pleasure and profit. A knowledge of tbe changes produced by the various processes of disease are essential not only to correct diagnosis, but to judicious treatment; to know what we cannot do is nearly as important as to know what can be done in the treatment of disease. A full knowledge of the minute processes of surgical disease may be learned from this book—it is unquestionably one of our most valuable works upon pathology.

*The Pharmaceutist's and Druggist's Practical Receipt Book, with a Glossary of Medical Terms, and copious Index.* By Thomas F. Branston. *Philadelphia:* Lindsay & Blakiston, 1865.

This work is eminently suited to the wants of the medical student, druggist, pharmaceutist and chemist, and is also valuable to the medical practitioner as a book of reference. It contains the pharmacopœal preparations, together with numerous miscellaneous receipts often useful in practice. Its value to the druggist and pharmaceutist is increased by a glossary of medical terms.

---

*A Monograph on Glycerin and its uses.* By Henry Hartshorne, A. M., M. D.

This is a full, complete, and highly instructive account of the properties and uses of glycerin. It is astonishing that an agent so long known should have been so poorly appreciated.

---

*Alphabetical Index to Braithwaite's Retrospect, embracing Parts one to fifty*—1840—1865.

This Index to Braithwaite's Retrospect is of inestimable value to those who have more or less complete numbers of this valuable work. It will be obtained by all who have the numbers and desire to make them available for reference.

---

*Literary Journals.*—The Atlantic Monthly and Godey's Lady's Book are placed regularly upon our table. These are standard works in polite literature, so to speak, and the publishers will please accept our thanks for the courtesy.

